# Risk Factors of Bone Nonfusion After Spinal Tuberculosis Debridement Bone Graft Fusion and Internal Fixation

**DOI:** 10.3389/fsurg.2022.888148

**Published:** 2022-05-18

**Authors:** Zihan Wei, Ying Zhang, Sizhen Yang, Jiawen Ye, Xu Hu, Tian Li, Tongwei Chu

**Affiliations:** ^1^ Department of Orthopedics, Xinqiao Hospital of Chongqing/Second Affiliated Hospital of Army Medical University, Chongqing, China; ^2^ School of Basic Medicine, Fourth Military Medical University, Xi’an, China

**Keywords:** spinal tuberculosis, postoperative bone nonfusion, risk factor, retrospective study, orthopeadics

## Abstract

**Objective:**

To retrospectively analyze bone graft nonfusion risk factors in spinal tuberculosis patients after lesion debridement, bone graft fusion and internal fixation.

**Methods:**

The clinical data of 131 patients who underwent spinal tuberculosis debridement, bone graft fusion and internal fixation in our hospital from March 2015 to March 2018 were retrospectively analyzed. The patients were divided into two groups according to bone fusion after the operation; there were 37 patients in the nonfusion group and 94 in the fusion group. The basic information and follow-up data of the patients were collected to evaluate the risk factors for bone graft nonfusion 1 year after surgery.

**Results:**

The severity of osteoporosis in the nonfusion group was significantly greater than that in the fusion group (*p* < 0.05). There were statistically significant differences between the two groups in terms of continuous multisegment status, disease duration, intraoperative surgical methods and whether patients received standardized drug treatment for 12 months after surgery (*p* < 0.05). Multivariate logistic regression analysis showed that long disease duration, posterior approach, and degree of osteoporosis were risk factors for postoperative bone graft nonfusion (OR > 1, *p* < 0.05), while standard drug treatment for 1 year after surgery was a protective factor (OR < 1, *p* < 0.05).

**Conclusion:**

Spinal tuberculosis patients who had a long disease course, who underwent simple posterior debridement, or who had severe osteoporosis had a higher risk of bone graft nonfusion after surgery. Tuberculosis treatment is beneficial for the osseous fusion of the postoperative bone graft area.

## Background

Tuberculosis is one of the most widespread infectious diseases in the world that occurs worldwide (1). According to researchers’ estimates, approximately one-quarter of the world’s population has been infected with pulmonary tuberculosis, and some tuberculosis infections can be found concomitantly outside the lungs. The most common type of extrapulmonary tuberculosis is bone tuberculosis with the spine being the most common site of bone tuberculosis, accounting for approximately half of all bone tuberculosis cases (2). At present, there are various options for the treatment of spinal tuberculosis, such as the use of standardized anti-tuberculosis drug therapy (3), but there are still some patients whose disease progresses after drug treatment, resulting in neurological damage and even paraplegia. Surgery is required when necessary (4). In the early stage, simple debridement or abscess drainage was often used to treat these patients (5), but these treatments could not improve the deformity or instability caused by the destruction of the spinal structure (6), so these methods have been gradually abandoned. Currently, spinal tuberculosis debridement and bone grafting, fusion and internal fixation are the most commonly used surgical methods for the treatment of spinal tuberculosis, and their efficacy in correcting kyphosis and relieving spinal cord and nerve compression has been widely recognized. One of the key aspects of these procedures is whether the implanted bone can be fused with the patient’s autologous bone, which will directly affect postoperative symptom relief and quality of life of the patient. Therefore, the risk factors for the complication of postoperative bone graft nonfusion deserve our close attention. In this study, the relevant data of patients who underwent spinal tuberculosis lesion removal, bone graft fusion and internal fixation in our hospital from March 2015 to March 2018 retrospectively analyzed, and the risk factors for bone graft nonfusion after spinal tuberculosis surgery were assessed. The findings and the suggestions for improvement of the treatment plan provide a relevant reference, and the report is as follows.

## Materials and Methods

### General Data

**Figure 1:**
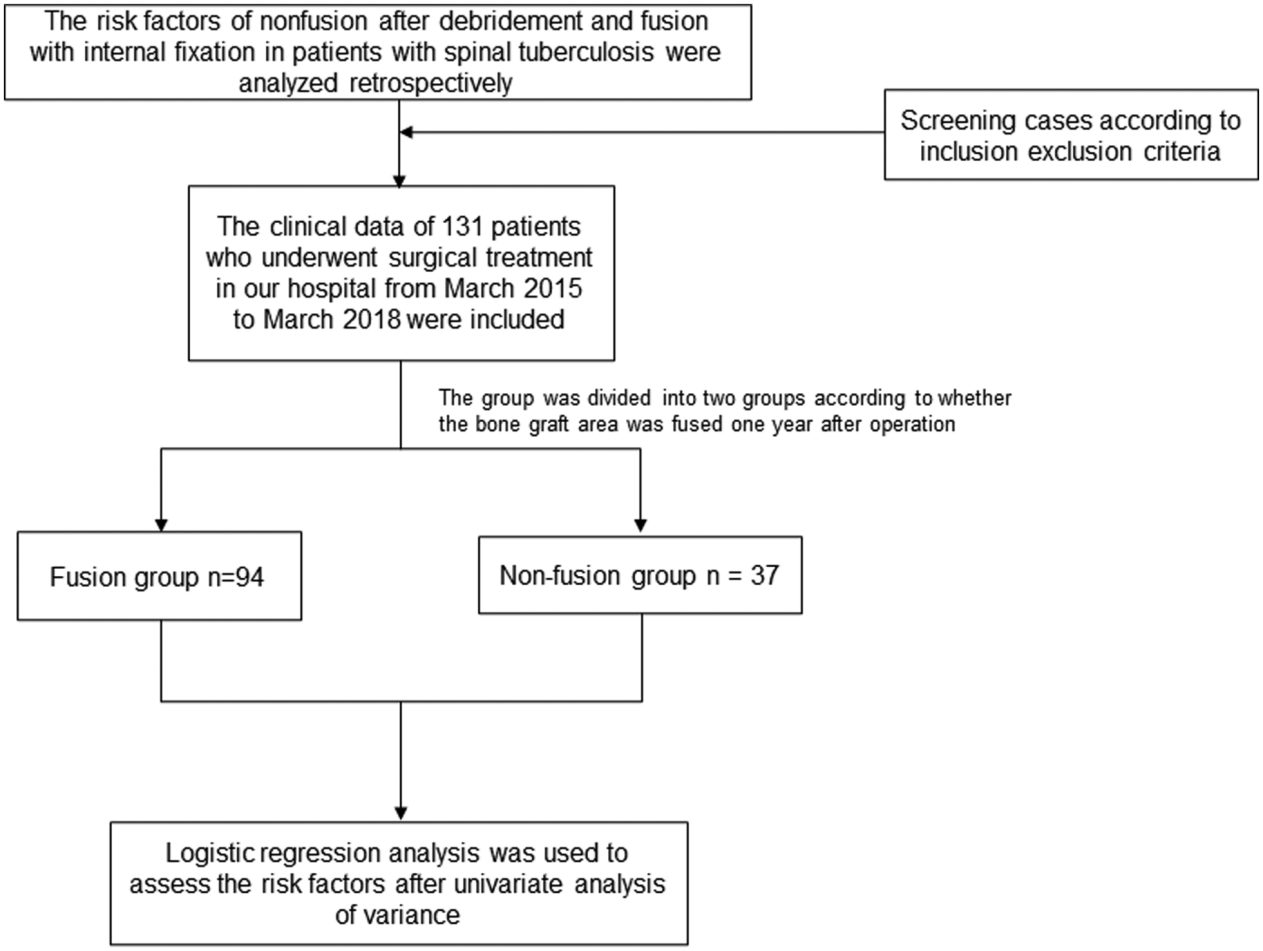
Study design and flow diagram of this article.

The study design can be found in Figure 1. The general data, surgical information and follow-up data of patients who underwent spinal tuberculosis debridement, bone grafting, fusion and internal fixation in our hospital from March 2015 to March 2018 were retrospectively analyzed. A total of 131 patients were included, including 58 females and 73 males. They were divided into a fusion group (94 cases) and a nonfusion group (37 cases) according to whether the bone fused one year after the graft (7). Whether bone fusion occurs according to the theory of Bridwell et al. They divided the graft fusion into four levels. Grade I: Fused with remodeling and trabeculae. Grade II: Graft intact, not fully remodeled and incorporated though; no lucencies. Grade III: Graft intact, but a definite lucency at the top or bottom of the graft. Grade IV: Definitely not fused with resorption of bone graft and with collapse (8). This study defines Grade I and Grade II as bone graft fusion. The general data of the two groups of patients, including sex, height, weight and body mass index (BMI), were not significantly different (*p* > 0.05) and were comparable. Regarding the severity of osteoporosis in the two groups (no osteoporosis/osteoporosis/severe osteoporosis), osteoporosis grading was based on the T value in the bone density test. T  ≥ −1 indicated no osteoporosis, −2.5 ≤ T <  −1 indicated osteoporosis, and T <   −2.5 indicated severe osteoporosis; the difference was statistically significant (*p *< 0.05). To rule out the effects of bone graft materials and ensure a higher fusion rate, all patients in this study were treated with autogenous bone graft. Declaration of Helsinki was followed throughout the study, and all patients were fully informed and signed informed consent before surgery.

### Inclusion and Exclusion Criteria

The inclusion criteria were as follows: ① patients with typical imaging features or clinical symptoms of spinal tuberculosis and postoperative pathological results clearly showing spinal tuberculosis; ② patient age <75 years old and ≥18 years old; ③ noninterrupted diseased segment; and ④ regular anti-tuberculosis treatment before operation, operative indications and normal operating conditions.

The exclusion criteria were as follows: ① concomitant spinal tuberculosis of the cervical and sacral spine; ② concomitant active tuberculosis in other parts; ③ concomitant insufficiency of important organs such as the heart, brain and kidney or serious diseases, which make the patient unable to tolerate drugs or surgery; and ④ Redo or revision spinal tuberculosis surgery.

### Clinical Data

Preoperative, intraoperative and postoperative follow-up data of patients were collected. The preoperative data were as follows: ① Local kyphosis angle: According to the patient’s preoperative X-ray film, the local kyphosis angle caused by spinal tuberculosis was measured and recorded. The included angle was that of the tangent to the upper edge of the vertebral body: a negative value represents lordosis, and a positive value represents kyphosis; ② Japanese Orthopaedic Association (JOA) score, including subjective symptoms (9 points), clinical signs (6 points), and daily activity limitation (14 points), which was used to evaluate human functional impairment; ③Visual analog scale (VAS) pain score (0 points represent no pain, 10 points represent the most severe pain that is unbearable), which was used for pain assessment; ④ Erythrocyte sedimentation rate (ESR) of preoperative venous blood test; ⑤ C-reaction protein (CRP) in venous blood test of patients before surgery; ⑥ Adjacent multisegment thoracic tuberculosis status (that is, whether the number of consecutive involved segments is greater than or equal to 3 segments); and ⑦ course of disease. The intraoperative data were as follows: ① surgical method (anterior debridement or posterior debridement); ② operation time; and ③ intraoperative blood loss. The postoperative follow-up data were as follows: completion of 1 year of standardized anti-tuberculosis drug treatment (drug treatment plan is isoniazid + rifampicin + ethambutol + pyrazinamide). Additionally, adverse reactions such as liver function damage and gastrointestinal reactions were monitored, and drug use was adjusted according to the patient’s condition, if the patient has a reasonable excuse to make a rational medication adjustment, we also believe that the patient is receiving standard medication.

### Statistical Processing

SPSS 25.0 was used for statistical analysis. The measurement data with a normal distribution are expressed as the mean ± standard deviation, and T test or analysis of variance were used for analyses. Measurement data conforming to a skewed distribution are expressed as the median (interquartile distance), and the rank sum test was used for analyses. The count data are expressed as frequencies and were analyzed by the χ^2^ test. The analysis of risk factors was based on the significant difference in single factor analysis, logistic regression analysis was used to analyze the risk factors, and *p* < 0.05 indicated a significant difference.

## Results

### Comparison of Two Sets of General Data

There were no significant differences in sex, height, weight or BMI between the two groups (*p* > 0.05). The severity of osteoporosis in the nonfusion group (no osteoporosis/osteoporosis/severe osteoporosis) was significantly higher than that in the fusion group (*p* < 0.05) Table 1.

**Table 1:** Comparison of two sets of general data.

	Fusion	Nonfusion	Statistics	*p* value
Sex (female/male)	42/52	16/21	0.022	0.881
Height (cm)	162.77 ± 7.29	165.11 ± 5.93	−1.721	0.088
Weight (kg)	57 (47–65)	63 (49–68)	−1.702	0.089
BMI (kg/m^2^)	21.29 (19.14–23.34)	22.06 (19.65–23.88)	−1.14	0.254
Osteoporosis severity	76/9/9	21/6/10	8.680	0.013

### Comparison of Preoperative Data Between the Two Groups

There was a statistically significant difference in continuous multisegment spinal tuberculosis and disease duration between the two groups (*p* < 0.05). The nonfusion group had more patients with continuous multisegment spinal tuberculosis, and the disease duration was longer. There was no significant difference in preoperative local kyphosis, JOA, VAS, ESR or CRP between the two groups (*p* > 0.05) (Table 2).

**Table 2:** Comparison of preoperative data between the two groups.

	Fusion	Nonfusion	Statistics	*p* value
Local kyphosis	20.15 (9.1–26.3)	11.8 (−4.6–18.3)	−1.825	0.068
JOA	18 (12–20)	19 (12–25)	−1.576	0.115
VAS	4 (4–4)	4 (4–5)	−0.923	0.356
ESR (mm/h)	55.97 ± 17.47	50.76 ± 15.811	1.578	0.117
CRP (µg/mL)	66 (47–95)	65 (25–84)	−1.284	0.199
Consecutive multisegment spinal tuberculosis (no/yes)	73/21	22/15	4.413	0.036
Course of disease (m)	7.5 (4–12)	22 (12–35)	−4.816	<0.001

### Comparison of Intraoperative and Follow-Up Data Between the Two Groups

There were significant differences in the surgical methods (anterior debridement/posterior debridement) and treatment with standardized anti-tuberculosis drug therapy for 1 year between the two groups (*p* < 0.05). There was no significant difference in operation time or intraoperative blood loss (*p* > 0.05) (Table 3).

**Table 3:** Comparison of intraoperative and follow-up data between the two groups.

	Fusion	Nonfusion	Statistics	*p* value
Surgical method (anterior/posterior)	61/33	13/24	9.566	0.002
Operation time (min)	221.99 ± 82.47	202.73 ± 67.13	1.264	0.208
Intraoperative blood loss (mL)	400 (350–600)	500 (300–600)	−0.449	0.654
1 year of standardized drug therapy (no/yes)	8/68	20/17	32.769	<0.001

### Logistic Multivariate Regression Analysis of Related Risk Factors

According to the results of univariate analysis and clinical experience, we selected the indicators of multivariate regression analysis and found that a long course of disease, the use of posterior debridement, and the severity of osteoporosis were risk factors for postoperative bone nonfusion (OR > 1, *p* < 0.05), and 1-year standardized anti-tuberculosis drug treatment was a protective factor (OR < 1, *p* < 0.05) (Table 4).

**Table 4:** Logistic multivariate regression analysis of related risk factors.

	B	SE	*p* value	OR (95% CI)
Continuous multisegment Spinal tuberculosis	0.511	0.538	0.342	1.667 (0.58–4.787)
Course of disease	0.023	0.011	0.038	1.023 (1.001–1.046)
Posterior debridement	1.037	0.51	0.042	2.822 (1.039–7.665)
Osteoporosis				
Light/none	1.106	0.729	0.129	3.023 (0.724–12.616)
Severe/None	1.847	0.684	0.007	6.339 (1.659–24.226)
One year of standardized drug therapy	−2.781	0.578	0	0.062 (0.02–0.192)

### Typical Images of Patients

**Figure 2:**
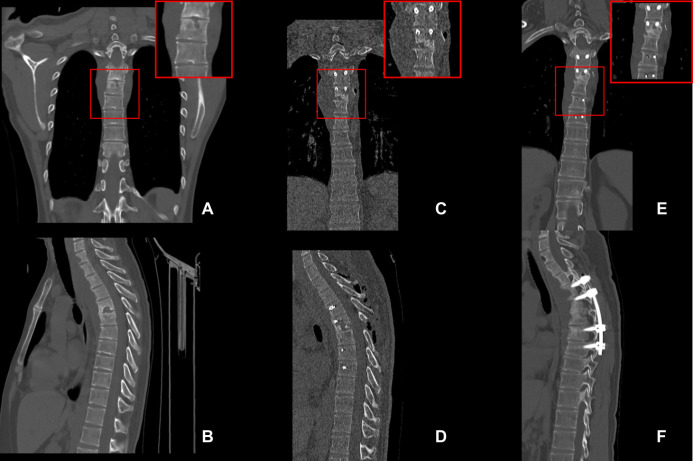
Images for selected typical case. A 32-year-old male patient was diagnosed with thoracic vertebral tuberculosis and underwent posterior spinal tuberculosis debridement, bone grafting, fusion, and internal fixation in our hospital (Figure 2). The patient had no osteoporosis and did not take medication regularly one year after surgery. (**A**/**B**) **A** are the preoperative CT scan of the thoracic spine showed bone destruction in the thoracic spine. (**C/D**) Postoperative CT scan of the thoracic spine showed that the bone graft exhibited good contact with the upper and lower vertebral bodies. (**E/F**) Plain CT scan of the thoracic spine at the 1-year follow-up. There is a clear boundary between the bone graft and the autologous bone, and the bone graft is not fused.

A 32-year-old male patient was diagnosed with thoracic vertebral tuberculosis and underwent posterior spinal tuberculosis debridement, bone grafting, fusion and internal fixation in our hospital (Figure 2). The patient had no osteoporosis and did not take medication regularly one year after surgery. A preoperative CT scan of the thoracic spine showed bone destruction in the thoracic spine. (**C/D**) Postoperative CT scan of the thoracic spine showed that the bone graft exhibited good contact with the upper and lower vertebral bodies. (**E/F**) Plain CT scan of the thoracic spine at the 1-year follow-up. It can be seen that there is a clear boundary between the bone graft and the autologous bone, and the bone graft is not fused.

## Discussion

Tuberculosis patients usually present with respiratory system damage, but some patients may have concomitant or simple extrapulmonary tuberculosis. Spinal tuberculosis, as one of the most common types of extrapulmonary tuberculosis (9), often leads to the destruction of vertebral bodies and intervertebral discs and even paravertebral abscesses. The early symptoms are radicular pain, local weakness, and hypoesthesia in the area innervated by the affected nerve, and severe cases may result in paraplegia (10). The current treatment options for spinal tuberculosis mainly include drug therapy and surgery. Regarding the use of anti-tuberculosis drugs as the basic treatment (11), when the treatment effect is not good or severe nerve damage and spinal deformity occur (12), surgery is the only option. An important way to treat spinal tuberculosis (13) is to maintain spinal stability through decompression and correction of local kyphosis (14), relieve the symptoms of patients, and improve the quality of life of patients. Spinal tuberculosis foci debridement and bone grafting, fusion and internal fixation, as some of the accepted surgical procedures for the treatment of spinal tuberculosis (15), have been widely implemented due to their advantages of short operation time, quick recovery, and low recurrence rate (16). However, there are postoperative complications, such as sinus formation, pleural or peritoneal injury, and nonfusion of bone grafts. Among these complications, nonfusion or delayed union of the bone graft has the most serious impact on the prognosis of the patient and may lead to subsidence of the diseased vertebra, displacement of the internal fixation or even fracture, which not only causes movement disorder of the normal motor unit but also may lead to the failure of the patient’s operation and reoperation.

In this study, we conducted a detailed assessment of the risk factors for bone nonfusion after spinal tuberculosis debridement, bone graft fusion and internal fixation based on relevant case data. Regarding the general information of the patients, there was no significant difference in sex, height, weight or BMI between the two groups (*p* > 0.05), and the two groups were comparable. The severity of osteoporosis (no osteoporosis/osteoporosis/severe osteoporosis) in the nonfusion group was significantly greater than that in the fusion group, and the difference was statistically significant (*p* < 0.05), which may be due to decreased density, defects in trabecular bone microarchitecture, and inherent defects in the material properties of bone tissue (17) resulting in poor bone healing. Additionally, osteoporosis may lead to the collapse of the intervertebral space, resulting in the loosening of the firm bone graft, which is more likely to cause nonfusion of the bone graft after surgery. This study found that among the two indicators of continuous multisegment spinal tuberculosis and disease duration, patients with continuous multisegment spinal tuberculosis and a longer disease course had a higher probability of postoperative bone nonfusion, and the difference was statistically significant (*p* < 0.05). Because spinal tuberculosis has the characteristics of affecting adjacent segments and spreading down ligaments, continuous multisegment spinal tuberculosis is often associated with more severe spinal cord injury and kyphosis than single-segment spinal tuberculosis (18), which also leads to the need to remove more bone to correct the deformity during surgery, possibly affecting the fusion rate of the bone graft to a certain extent. The length of the disease course can also reflect the severity of the spinal damage to a certain extent. Patients with a longer disease course tend to have a wider range of abscesses and more serious bone destruction, which will also affect surgical debridement and the bone grafting fusion effect. There was no significant difference in preoperative local kyphosis, JOA, VAS, ESR or CRP between the two groups (*p* > 0.05). Multivariate regression analysis also confirmed that the severity of osteoporosis and the length of the disease course were independent risk factors for postoperative bone graft nonfusion.

At present, there are two main approaches for the debridement of spinal tuberculosis lesions: anterior debridement and posterior debridement (19). Since spinal tuberculosis lesions are usually located in the anterior column, the advantage of anterior debridement is that the lesions can be directly exposed. For spinal tuberculosis lesions, more thorough debridement and spinal cord decompression can be performed, and bone grafting can be performed in a better surgical field when performing bone grafting. However, anterior exposure of the upper thoracic region is difficult due to the anatomic characteristics of the anterior approach, so it is sometimes not suitable for patients with multisegmental thoracic vertebral involvement (20). The results of this study revealed that there were significant differences between the two groups in terms of the surgical method (anterior debridement or posterior debridement) and whether the patient received standardized drug therapy for 1 year after surgery (*p* < 0.05). There was no significant difference in operation time or blood loss between the two groups (>0.05). Posterior debridement will reduce the rate of postoperative bone graft fusion, but according to the surgical risk and trauma of patients, the choice of surgical method should still vary from person to person, and the anterior approach should not be insisted on to ensure bone graft fusion because it could lead to an increased risk of other complications. As the basis of all treatments, drug therapy for spinal tuberculosis should be used in a standardized manner before and after surgery. According to some data, drug therapy can improve fusion after spinal tuberculosis surgery (3); therefore, postoperative standardized drug therapy should not be ignored. Multivariate regression analysis also confirmed that posterior debridement was an independent risk factor for bone graft fusion, and the completion of one-year postoperative standardized drug therapy was a protective factor for bone graft fusion. This study has the limitation that the number of cases was somewhat small, and we will continue to improve and expand upon this study in follow-up work.

In summary, although most patients with spinal tuberculosis can achieve good symptom relief and stable bone graft fusion after surgery, patients with more severe osteoporosis, a longer disease course, or posterior debridement surgery may have a significantly higher risk of postoperative bone graft nonfusion or delayed fusion than other patients. Completion of standardized anti-tuberculosis drug therapy after operation can reduce the risk of nonfusion of bone grafts to a certain extent. The results of this study can provide a more systematic and comprehensive treatment plan and reference for spine surgeons in the surgical treatment of spinal tuberculosis.

## Data Availability

The original contributions presented in the study are included in the article/Supplementary Material, further inquiries can be directed to the corresponding author/s.
